# Bony callus stiffness indirectly evaluated by the axial load-share ratio in vivo as a guide to removing a monolateral external fixator safely

**DOI:** 10.1007/s00264-021-05116-z

**Published:** 2021-06-23

**Authors:** Yanshi Liu, Feiyu Cai, Kai Liu, Xingpeng Zhang, Hong Li, Xuefei Fu, Tao Zhang, Aihemaitijiang Yusufu

**Affiliations:** 1 Department of Trauma and Microreconstructive Surgery, the First Affiliated Hospital of Xinjiang Medical University, Urumqi, Xinjiang China; 2 Department of Orthopedics, Shanghai Pudong New Area People’s Hospital, Shanghai, China; 3 Department of Orthopedics, Zigong Fourth People’s Hospital, Zigong, Sichuan China; 4 Department of Orthopedics, Anhui No. 2 Provincial People’s Hospital, Hefei, Anhui China; 5 Department of Orthopedics and Trauma, Tianjin Hospital, Tianjin, China

**Keywords:** Axial load-share ratio, Bony callus stiffness, Fracture healing, Monolateral external fixator, Timing of fixator removal

## Abstract

**Purpose:**

As the monolateral external fixator is increasingly used in trauma-control and definitive management for high-energy long bone fractures, timing the fixator removal remains a challenge for surgeons. The purpose of this study was to determine the feasibility and effectiveness of the bony callus stiffness indirectly evaluated by the axial load-share ratio in vivo as a guide to removing a monolateral external fixator safely.

**Methods:**

A total of 131 patients with tibial shaft fractures treated by the monolateral external fixator in our institution were collected from January 2013 to July 2019. In group I, the fixators were removed based on the clinical and radiological assessment only by the treating surgeon. As for group II, the axial load-share (LS) ratio test was accomplished by another medical team without the knowledge of the clinical results. The external fixator was removed when the mechanical test outcome (LS ratio < 10%) was consistent with the conclusion drawn from the clinical and radiological assessment (bone union achieved) by the treating surgeon.

**Results:**

There was no statistical significance in demographic data between the two groups (P > 0.05). In group I, four patients suffered refracture (the refracture rate was 7.7%) after fixator removal and were successfully treated by an intramedullary nail. In group II, 71 patients underwent fixator removal after the first mechanical test, and another eight patients terminated the external fixation after the second test. None of the 79 patients in group II suffered refracture (the refracture rate was 0%). There was statistical significance in the refracture rate between the two groups (P < 0.05).

**Conclusion:**

The bony callus stiffness indirectly evaluated by the axial load-share ratio in vivo using the additional circular frame components is an effectively quantitative indicator to complement the clinical assessment of fracture healing in a monolateral external fixation treatment. Removal of the monolateral external fixator is safe when the axial load-share ratio dropped below 10%.

## Introduction

The external fixation acts a crucial role in the management of infected bone nonunion, complex extremity deformity, bone defects caused by various injuries, and high-energy fractures where internal fixation is impossible [1–5]. For fractures treated by external fixation, the timing of fixator removal is a problem worth considering. External fixators are wished to be removed as early as possible for most patients due to the discomfort in wearing. Early removal of the external fixator introduces the risk of deformation or refracture, but complications are increased if removal is delayed.

The fracture union is generally defined as the reconstruction of the bony biomechanical characteristic. However, the bone union is traditionally evaluated using imaging modalities, such as conventional radiographs in two planes, dual-energy X-ray absorptiometry (DEXA), computed tomography (CT), magnetic resonance imaging (MRI), and ultrasound (US) [6–11]. Although the aforementioned methods are commonly applied in clinical practice, neither of them provides related biomechanical information. It is challenging to determine the original biomechanical feature is achieved at the fracture site.

Thus, the exploration of a biomechanical technique to monitor the bone healing process is gaining growing attention. Aarnes et al. developed a device equipped with tension–compression load cells and included a mathematical analysis to assess the axial stiffness of the regenerate tissue in vivo[12]. The external fixator was removed when the load-share (LS) ratio dropped below 10% in their clinical trial of 22 patients who underwent tibial lengthening with Ilizarov circular external fixator, and none suffered refracture after removing the frame. Their study was derived from the theoretical basis of an externally applied load is shared between the fixator and the regenerating bony callus, and the load carried by the bony callus depends on its stiffness which will increase with mineralization.

As the monolateral external fixator is increasingly used in trauma-control and definitive management for high-energy long bone fractures, timing the fixator removal remains a challenge for surgeons. Therefore, in the present study, the method of Aarnes et al. [12] was transformed and applied to the removal of a monolateral external fixator. The purpose of this study was to show the feasibility and effectiveness of the bony callus stiffness indirectly evaluated by an axial load-share ratio in vivo for assessing the fracture healing, and to determine if the load-share (LS) ratio dropped below 10% could be used as a safe limit to remove a monolateral external fixator.

## Materials and methods

In the first stage of the present study, we retrospectively collected a consecutive series of 73 patients (52 eligible patients) (group I) with tibial shaft fractures treated by the monolateral external fixator in our institution, from January 2013 to April 2015, including 45 males and seven females with a mean age of 40 years (range 24 to 61 years). The external fixator removal was depended on the radiological and clinical assessment by the treating surgeon.

Subsequently, we conducted a prospectively observational study in a consecutive series of 112 patients (79 eligible patients) (group II) who were admitted to our institution and consented to definitive monolateral external fixation treatment for tibial shaft fractures from August 2015 to July 2019, including 67 males and 12 females with a mean age of 39 years (range 18 to 62 years). According to the mathematical analysis and clinical conclusion of Aarnes et al. [12], when the conclusion that drawn from the mechanical test by another medical team (LS ratio < 10%) is consistent with the radiographs (bridging callus appeared) and clinical assessment (bone union achieved), the external fixator was dynamized and removed later.

All the 131 patients were treated by the same surgical team. The monolateral external fixation treatment was performed due to trauma-control and definitive management for open fractures or closed fractures with poor surrounding soft tissues. Fractures with crucial vascular and nerve injury, pathological fractures, age > 65 years, poor compliance, pin loosening, and any other illness that can affect bone healing (such as diabetes, osteoporosis, kidney disease) were excluded. Informed consent was obtained from all patients for their data to be documented and published in the present study. This study was approved by the Ethical Committee of our institution.

### Principles of mechanical measurement

**Fig. 1:**
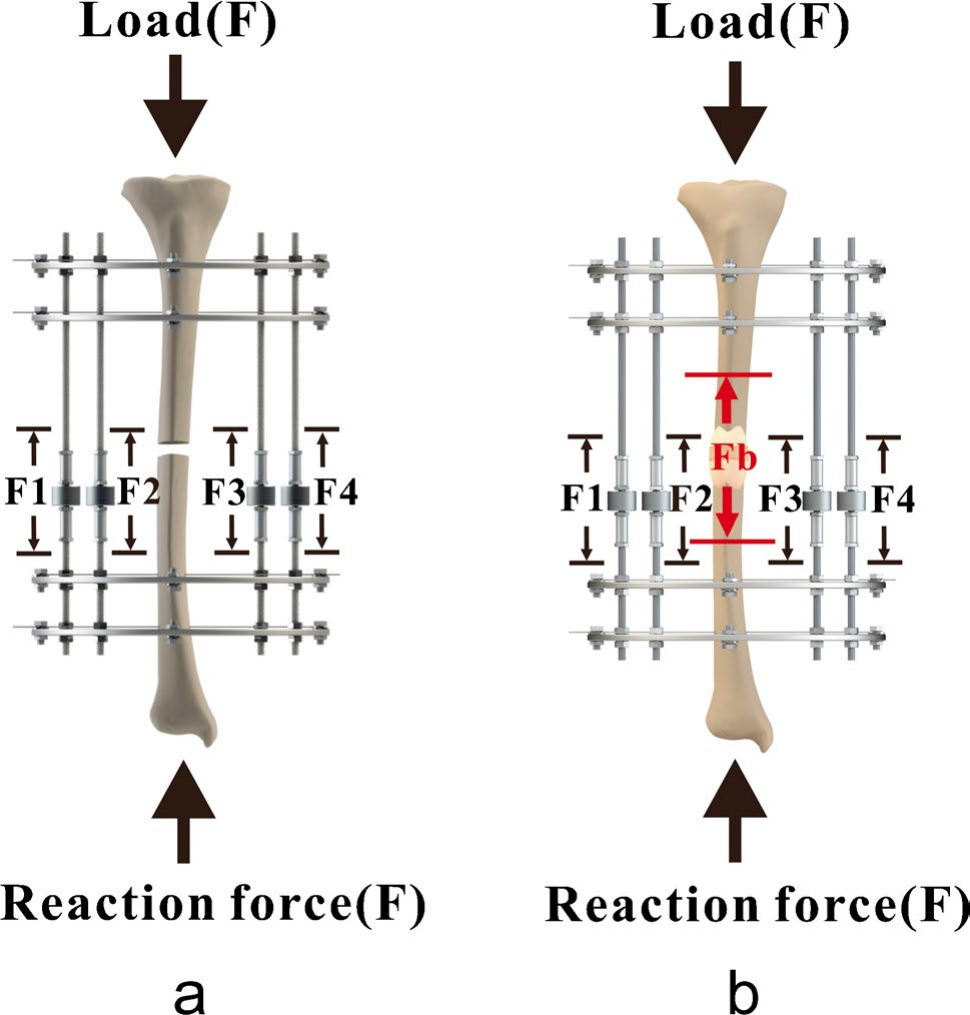
Schematic diagram of load sharing. F is the entire force applied externally on the injured limb. Fb is the load carried by the bony callus. F1, F2, F3, and F4 are the load shared by each fixator rods. **a** All loads are carried by the fixator in the early phase of healing. **b** With the callus in progressive mineralization and gradually stiffens, more load is carried by the bone leading to a reduced load at the fixator

The load carried by the bony callus is determined by its stiffness. The load-share ratio definition is the compressive force in the fixator rods divided by the known applied external load [12]. At the beginning of fracture healing, the bony callus stiffness is zero, the external load is therefore entirely borne by the external fixator, and the LS is 100% at this stage (Fig. 1a). With the callus in progressive mineralization and gradually stiffens, the load shared by the new bone is increase, and the LS ratio is decrease conversely (Fig. 1b). Hence, the load-bearing capacity of the regenerate can be indirectly and objectively reflected by the load-share ratio.

According to the previously published data [12], the known externally applied load (F) is shared between the fixator rods (F1, F2, F3, and F4) and the bony callus (Fb). The complete load carried by the fixator is defined as the sum of load undertaken by each rod. (Fig. 1).

In a simplified model, the definition of LS is

1 $$LS=\frac{F1+F2+F3+F4}{F}$$

### Devices for load measurement

**Fig. 2:**
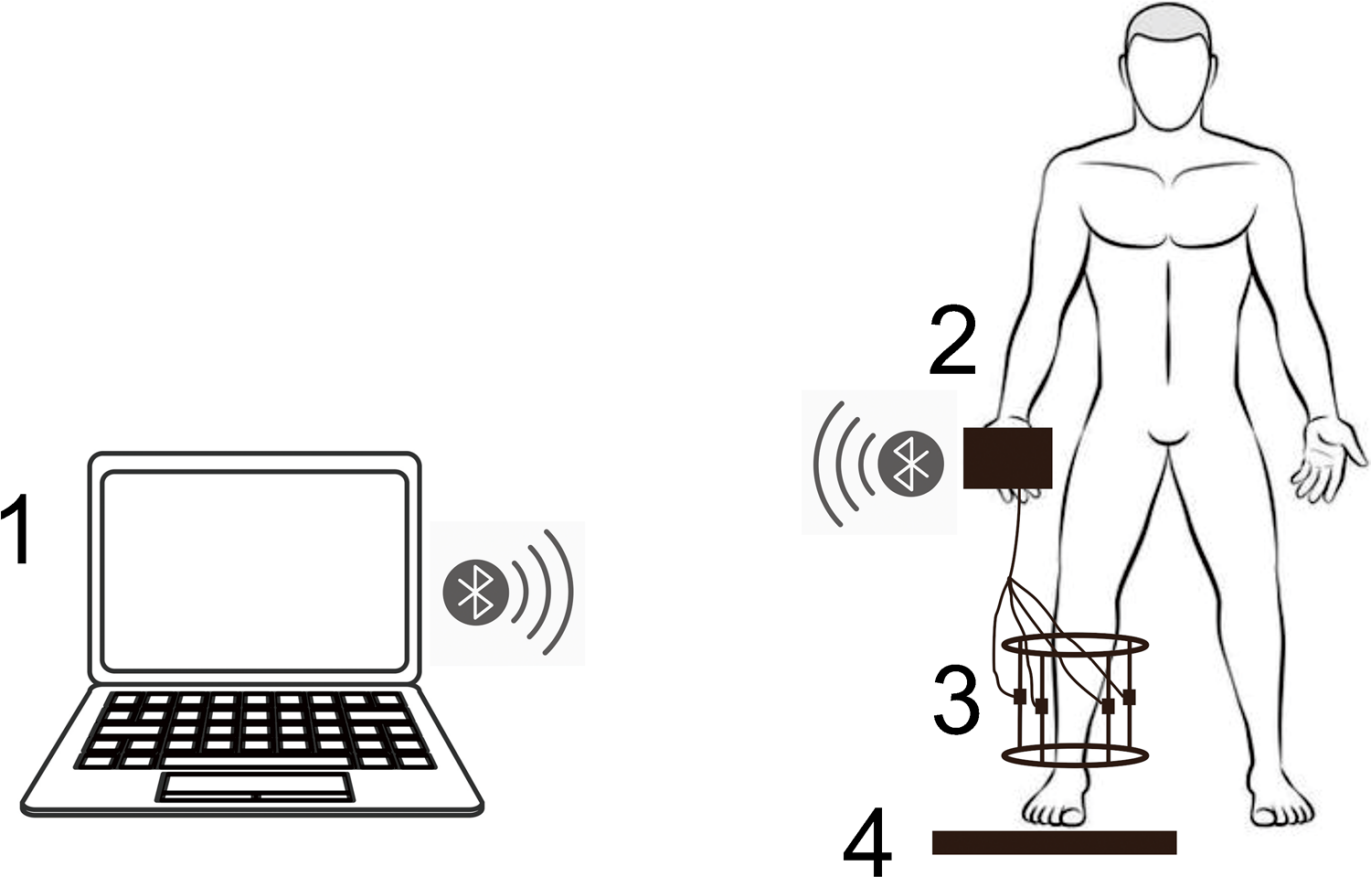
Schematic diagram of the test procedures and total devices. The mechanical signals are transmitted wirelessly by Bluetooth technology. **1** Computer. **2** A/D converter. **3** Tension–compression force sensors. **4** Force platform

**Fig. 3:**
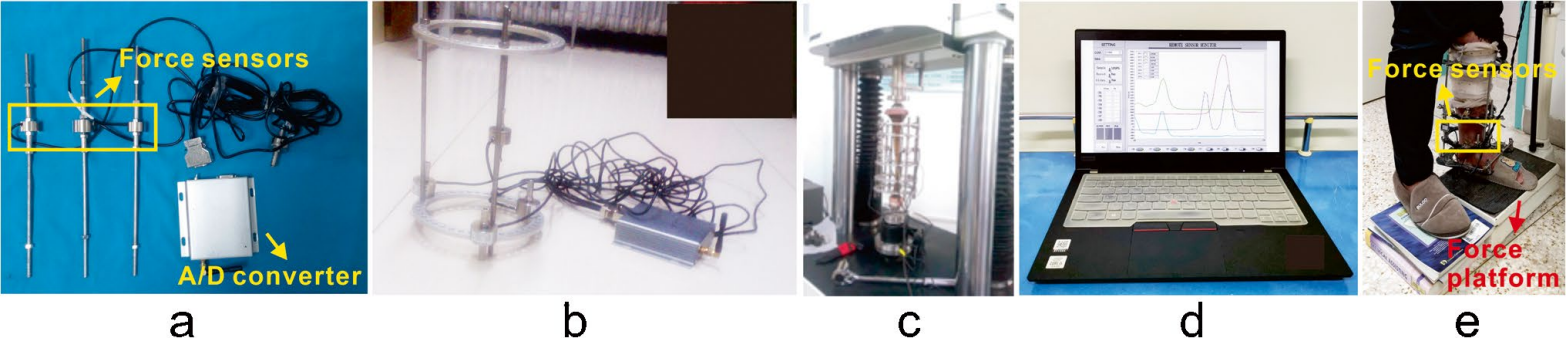
Devices for load measurement in the present study. **a** Force sensors and A/D converter. **b** General picture of the installation. **c** The force sensors were calibrated by a material test machine, as well as the effectiveness of the whole device. **d** A customized computer software. **e** A force platform for externally applied load assessment

Three bars are sufficient to keep the frame stable. The complete device for load measurement consists of three fixator rods with a dismountable force sensor (maximum load of 500 N, HYLY-019, Bengbu Hengyuan Sensor Technology Co., China) on each rod, a custom-made A/D converter, a force platform (maximum load of 1200 N, RGZ-120, Jiangsu Suhong Medical Instrument Co., China), and a customized computer software. The force sensors are used to measure the load shared by the fixator; the output signals are wirelessly transmitted to the computer through the A/D converter. The externally applied load is equal to the reaction force and measured by the force platform. The mechanical data were recorded and analyzed by the customized computer software. The force sensors were calibrated by a material test machine (BOSE Electroforce 3150, USA), as well as the effectiveness of the whole device (Fig. 2 and Fig. 3).

### Procedures of clinical application

When sufficient union was achieved in the fracture site evaluated by the treating surgeon, the mechanical test was performed by another medical team without the knowledge of the clinical results.

**Fig. 4:**
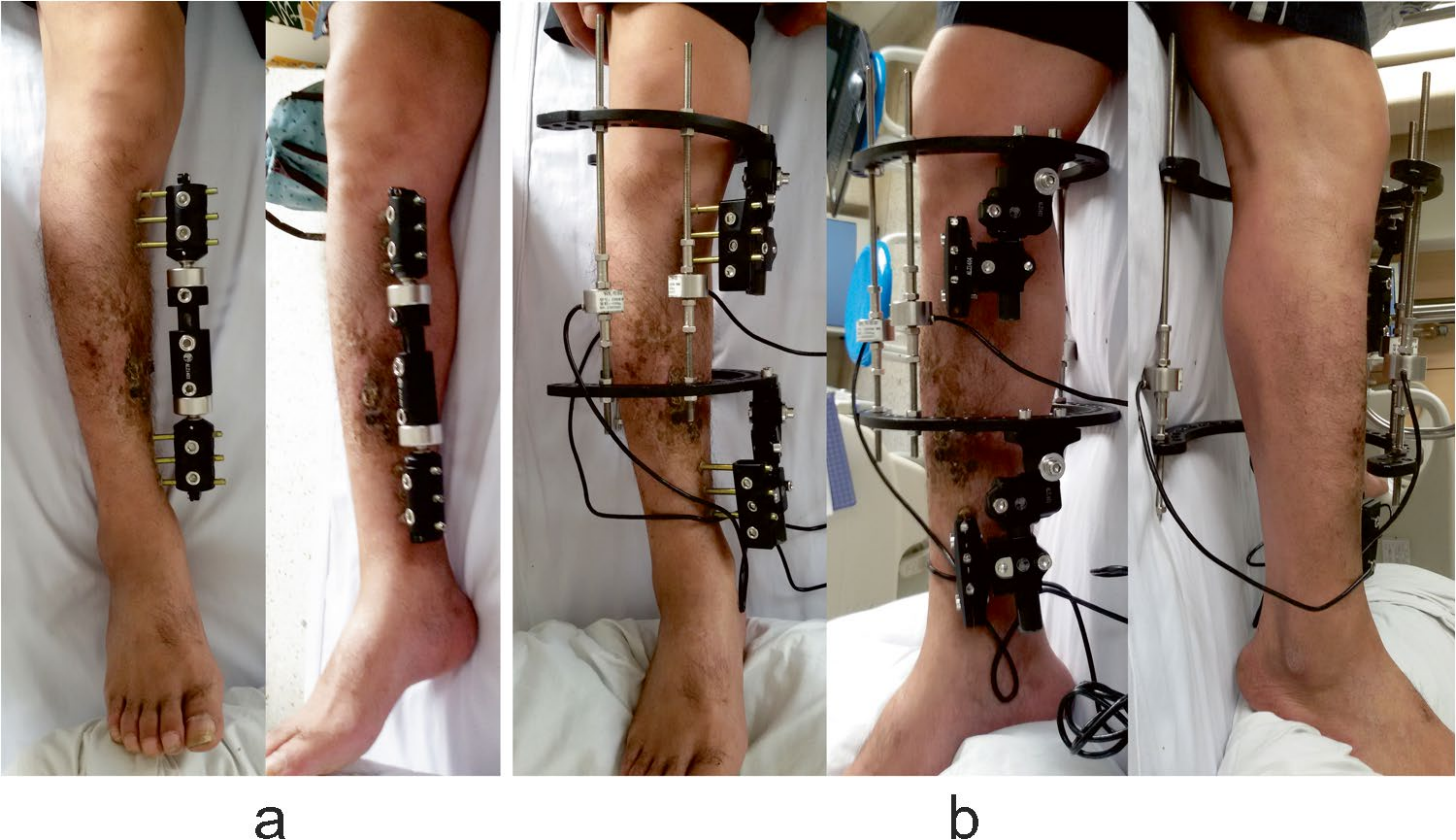
Installation of the devices in a patient treated by a monolateral external fixator. **a** General pictures before installation. **b** Successful installation with the help of external fixation components

During the test procedures, the original connecting rod of the monolateral external fixator was removed firstly, taking care to retain the original half pins. Subsequently, two circular or partial rings were temporarily installed on these pins with the help of external fixation components. The two rings were attached to each bony segment and were perpendicular to the long axis of the injured bone in an orthogonal manner. The force sensors are attached to three rods and then connected force-free between the two rings in the fixator. It is crucial to ensure that the three bars are parallel by adjusting the cardan shaft of the external fixation components. The measuring bars, therefore, took over the fixator load completely during the measurements, including inherent stresses of the bone–soft–tissue-fixator mounting (Fig. 4).

Static test was conducted in the present study, loading the limb with a known force (full weight bearing or external compressive force within 300 N, according to our usual clinical practice), and the investigator must take care that the patient was relaxed during the procedures to minimize the effect of muscle activity. The force sensors were only required to be zeroed before the test without the need to be calibrated each time, and then the carried load is saved and appeared by the computer software. The evaluations were performed with the Excel spreadsheet program. For an accurate measurement, three static tests must be performed to obtain the mean valid forces.

### Management after fixator removal

All patients were warned to use the limb only as much as necessary and report any adverse events after fixator removal. Clinical visits and radiographs were routinely taken two weeks later.

### Statistical analysis

Statistical analysis was performed with the SPSS 22.0 (IBM Corp, USA). Continuous variables were analyzed by independent-samples T-tests and expressed as the mean and range. The count variables were analyzed by the chi-square or Fisher’s test, expressing as number. A statistically significant difference was set at P < 0.05.

## Results

The details of the two groups are shown in Table 1. There was no statistically significant difference in demographic data between the two groups (P > 0.05). Statistical significance was observed in the refracture rate between the two groups (P < 0.05).

**Table 1:** Details of patients in the two groups

	Group I	Group II	Statistical value	P value
Mean age in years (range)	40 (24 to 61)	39 (18 to 62)	1.021	0.309
Gender (male:female)	45:7	67:12	0.076	0.783
Fracture type (AO classification)				
Type A	31	49	1.021	0.600
Type B	16	26		
Type C	5	4		
Open/closed fracture				
Open	13	19	0.015	0.902
Closed	39	60		
Gustilo’s classification				
Type I	4	6	-	1.000
Type II	7	10		
Type IIIA	2	2		
Type IIIB	0	1		
Mean external fixation time in weeks (range)	25 (18 to 36)	26 (18 to 39)	− 1.106	0.271
Mean time of follow-up in months (range)	16 (12 to 24)	15 (12 to 25)	1.453	0.149
Refracture rate	7.7% (4/52)	0% (0/79)	3.939	0.047

In the first group (group I), the mean external fixation time to bone union was 25 weeks (range 18 to 36 weeks). Four patients of 52 suffered refracture after fixator removal and the refracture rate was 7.7%. The four refractures occurred slowly in a period of time and resulted in angular displacement. Further intervention was performed by intramedullary nail, and bone union was finally achieved. The 52 patients were successfully followed at an average of 16 weeks (range 12 to 24 weeks).

In the mechanical test group (group II), none felt any discomfort during the testing procedures. The mean duration of testing procedure was 16.8 minutes (range 14 to 23 min). Seventy-one patients achieved axial load-share ratio below 10% (range 0.7 to 9.6%) at the first mechanical test and underwent fixator removal. However, another eight patients (group II) showed an axial load-share ratio that exceeded 10% (range 10.5 to 14.3%) at the first test and then continually treated by external fixation. The mean time elapsed from initial monolateral external fixation to the first mechanical test was 25 weeks (range 19 to 30 weeks). After a mean time of four weeks (range 3 to 5 weeks), all their mechanical data were dropped below 10% (range 3.1 to 8.2%) at the second test, and the external fixation treatment was therefore terminated. The mean external fixation time of these eight patients was 28 weeks (range 24 to 35 weeks). The average total external fixation time of the 79 patients was 26 weeks (range 18 to 39 weeks) (Details are shown in Table 2).

**Table 2:** Details of patients who underwent two mechanical tests in group II

Case	Gender	Age (yr)	Fracture type (open or closed)	First time (wk)	First LS ratio (%)	Second time (wk)	Second LS ratio (%)
1	Male	48	A3 (closed)	28	14.3	33	8.2
2	Male	27	B2 (closed)	21	12.5	25	6.5
3	Male	43	B1 (closed)	26	11.8	29	5.6
4	Male	38	A2 (closed)	22	13.2	27	7
5	Male	24	B2 (closed)	19	12.6	24	4.8
6	Female	52	A1 (open)	30	14	35	7.2
7	Male	35	B1 (closed)	23	13.8	27	5.3
8	Male	43	B2 (closed)	27	10.5	30	3.1

First time: Time elapses from initial external fixation to the first mechanical test

Second time: Time elapses from initial external fixation to the second mechanical test

*LS ratio*, load-share ratio

The mean follow-up in group II after the monolateral external fixator removal with an axial load-share ratio less than 10% was 15 months (range 12–25 months), and none of the patients suffered refracture.

Pin tract infection was the most common complication, as expected. Thirty-nine patients suffered superficial pin tract infection and successfully managed by oral antibiotics and meticulous pin site care. Intravenous antibiotics and pin replacement were used for the three cases with deep pin tract infection. None developed to sequestrum requiring surgical intervention.

## Discussion

With the increasing number of high-energy complex fractures, the external fixator has become an essential device in trauma centres, including the advantages of simple, minimally invasive, and can preserve the biomechanical microenvironment needed for bone healing [13]. During the external fixation treatment, timing fixator removal is an important decision for a satisfactory outcome. Leaving the frame longer than necessary would lead to various complications, while premature removal of the frame could result in angle displacement deformities or refracture. Ilizarov himself also remarked that “leaving the apparatus on for longer than necessary is as harmful as removing the fixator too early” [14].

The removal of external fixation was traditionally performed on the basis of clinicians’ clinical evaluation and the radiographic appearance of the callus [15]. However, the imaging techniques lack the biomechanical and quantitative information about the bony callus, and their accuracy in determining fracture healing has been questioned. The previous study has shown that radiographic evaluations are subjective and inaccurate [16], and there is no correlation between the callus amount and the healed bone stiffness [17]. The bone healing results are poorly predicted by both the general appearance and cortical bridging. Therefore, the radiographs provide limited guidance for removing the external fixator.

Fracture healing is a complex process, depending on various biological and mechanical factors. The skeleton is a load-bearing structure. Resistance to deformation is a fundamental property of a structure and is defined as its stiffness, which seems to be an appropriate assessment for bone regenerate. As fracture healing progresses in external fixation treatment, a steady increase in strength and stiffness, the bony load bearing properties will regain at some stage. At this point, the external fixation becomes redundant, and this moment is called the “healing endpoint.” Goodship et al. [18] also proved an increase in stiffness and stability of regenerated bone after fracture healing during time progression. Knowledge about the regenerate bone’s stiffness is therefore essential for judging the safe time to remove the external fixator.

Defining the endpoint for fracture healing is difficult, while the determination of a time point at which fracture healing is complete may be helpful for clinical decisions. Information on callus stiffness provided a good measure of healing [19]. Several techniques have been developed to quantify bone healing in mechanical terms. For callus stiffness obtained directly, most studies determine the callus stiffness by measuring the deflection of the healing bone under loading. Jerngerger [20] had described a method for obtaining an objective measure of the stability of tibial shaft fracture during bone healing in 1970. Jorgensen [21] illustrated the terminal healing phase in 35 crural fractures treated with the Hoffmann apparatus by systematically measure the bone deflections during load bending. Hammer et al. [22] ascertained fracture union time and the strength are sufficient for full weight-bearing without protection in 157 patients using this noninvasive method. The safe limit of callus stiffness ranged from 8.5 to 20 Nm/degree in these studies above. Subsequently, Richardson [23] defined a limit for removing an external fixator by measuring fracture stiffness in 212 patients with tibial fractures treated by external fixation. They considered that stiffness of 15 Nm/degree in the sagittal plane provides a useful definition of the union of tibial fractures. Wade et al. [24] complemented this indicator, advocating that fracture stiffness should be measured in two orthogonal planes and suggesting that values above 15 Nm/degree in two planes indicate to remove the fixator safely. Although accurate estimation is obtained, this direct method is limited by the removal of the fixator for each measurement. Furthermore, in the premature phase of bone healing, there is a potential risk of losing the reduction under bending load.

For clinical practicality and security, the deformation in the longitudinal axis of the bone was usually assessed. Most published studies described an indirect measurement based on the load that applied to the injured extremity is shared between the bone and fixator. This load sharing depends mainly on the biomechanical characteristic of the regenerate callus. In the early phase of bone healing, the load is mainly carried by the external fixator due to the fracture gap. The bony callus will increase in diameter and stiffness because of progressive calcification, resulting in an increasing carried load for the callus. With the increasing healing time, this will lead to a reduced load carried by the fixator. Evans et al. [25] developed a transducer that been fitted to the support column of an external fixator to monitor the fracture healing for terminating the external fixation. Aarnes et al. [12] described a mathematical model and evaluated the callus stiffness using axial load-share ratio in vivo, concluding that the external fixator can be safely removed when the load-share ratio dropped below 10%.

In the present study, we transformed the method of Aarnes et al. and applied it to a monolateral external fixator. The injured bone underwent axial load instead of bending load, and there is rarely a potential risk of reduction loss. The axial load-share test was conducted in a consecutive of 79 patients (group II) with tibial shaft fractures treated by a monolateral external fixator. With a mean of 15-month follow-up, there was none experienced refracture after removing the external fixator with an axial load-share ratio dropped below 10%. Refracture after removing the external fixator was one of the few major complications reported by De Bastiani [26], affecting 3% of patients, as well as Fischgrund et al. [27]. Others have reported rates of 6% [28] and 9.4% [29]. Compared with the mechanical test group, the refracture rate in group I was 7.7% in this study. There was statistical significance in the refracture rate between the two groups. The clinical results also manifested that the bony callus stiffness indirectly evaluated by the axial load-share ratio in vivo as a supplemental guide to evaluate the fracture healing made the fixator removal safer.

There was a group of eight patients that the treating surgeon had decided to remove the fixator, but the mechanical result has overruled this decision at the first test in this study. After a mean time of four weeks, the external fixator was safely removed based on the axial load-share ratio dropped below 10%. We speculate that the appearance of cortical bridging in radiographs lacks real mechanical information. Even if the bone union was seen on the radiographic modalities, the biomechanical properties of the regenerate tissue itself might not have thoroughly recovered. The mean external fixation time to union was 25 weeks in group I, while 26 weeks in group II. Although the measurement of fracture stiffness seems slightly postponed the mean external fixation time, there was no statistically significant difference (P > 0.05).

A low LS ratio may be caused by a small bone bridge that shares a significant load without the complete bone healed. We, therefore, suggest that radiographs must be taken to evaluate the geometry of the regenerate bone for the prevention of inaccurate mechanical information. Given that both the force platform and the force sensors are sensitive to axial load only, there will be inaccurate measurements due to the shear forces as well as torsion and bending moments in the monolateral fixator caused by the possible angle between the bars and long axis of the injured bone. To resolve this problem, we also recommend that the two additional rings should be perpendicular to the long axis of the injured bone in an orthogonal manner and ensure that the three bars are parallel with each other.

This noninvasive mechanical technique provides an objective and quantitative assessment of fracture healing, including potential advantages of reducing the number of radiographic images taken (lower cost) and a lower dose of ionizing radiation absorbed by the patient. The most important part of the whole device is the dismountable force sensor which is inexpensive and easy to get, and the other components can be easily obtained from the already used external fixator parts. The total device is price-friendly and manufacture-simply. This technique also does not involve complex testing procedures of excessive duration (mean duration was 16.8 min in this study) and electronic devices that remain for a long time or even forever in patients. After a brief training with a short learning curve, general orthopaedic surgeons are equal to finish this work. There are chances for its wider use in most fracture clinics.

The present study had several limitations. Firstly, considering its relatively small sample size, a prudent attitude should be adopted to interpret the potential greater risk of refracture if the fixator was removed based on clinical assessment only. Secondly, other tests are needed to determine whether there is a superior limit of LS ratio to assess the callus stiffness in the subsequent study. Thirdly, the forces acting on the fracture site will be influenced by the muscle activity with no control, and the loose fixator pins can disturb the reliability of the mechanical test. These problems are the source of inaccuracy in the mechanical test. Fourthly, this device can only be used in fractures treated with an external fixator. Despite these potential obstacles, the axial load-share ratio supplements the traditional clinical assessment and makes us safe while removing the external fixator.

## Conclusion

A comprehensive evaluation of fracture healing is crucial before removing the external fixator. The bony callus stiffness indirectly evaluated by the axial load-share ratio in vivo using the additional circular frame components is an effectively quantitative indicator to complement the clinical assessment of fracture healing in a monolateral external fixation treatment. Removal of the monolateral external fixator is safe when the axial load-share ratio dropped below 10%.

## Data Availability

The datasets analyzed during the current study are available from the corresponding author on reasonable request.
